# Epithelial derived CTGF promotes breast tumor progression via inducing EMT and collagen I fibers deposition

**DOI:** 10.18632/oncotarget.4659

**Published:** 2015-07-29

**Authors:** Xiaoping Zhu, Jing Zhong, Zhen Zhao, Jianting Sheng, Jiang Wang, Jiyong Liu, Kemi Cui, Jenny Chang, Hong Zhao, Stephen Wong

**Affiliations:** ^1^ Department of Systems Medicine and Bioengineering, Houston Methodist Research Institute, Weill Cornell Medical College, Houston TX, USA; ^2^ Department of Radiology, The Teaching Hospital of Fujian Medical University, Fujian Provincial Cancer Hospital, Fuzhou, China; ^3^ Department of Radiology, Zhongda Hospital, Nanjing, China; ^4^ Department of Orthopedics, Tongji Hospital, Wuhan, China; ^5^ Department of Pharmacy, Changhai Hospital, Shanghai, China; ^6^ Methodist Cancer Center, Houston Methodist Hospital, Houston TX, USA

**Keywords:** connective tissue growth factor (CTGF), epithelial-mesenchymal transition (EMT), collagen I fibers, tumor necrosis factor receptor 1 (TNFR1) pathway, breast cancer

## Abstract

Interactions among tumor cells, stromal cells, and extracellular matrix compositions are mediated through cytokines during tumor progression. Our analysis of 132 known cytokines and growth factors in published clinical breast cohorts and our 84 patient-derived xenograft models revealed that the elevated connective tissue growth factor (CTGF) in tumor epithelial cells significantly correlated with poor clinical prognosis and outcomes. CTGF was able to induce tumor cell epithelial-mesenchymal transition (EMT), and promote stroma deposition of collagen I fibers to stimulate tumor growth and metastasis. This process was mediated through CTGF-tumor necrosis factor receptor I (TNFR1)-IκB autocrine signaling. Drug treatments targeting CTGF, TNFR1, and IκB signaling each prohibited the EMT and tumor progression.

## INTRODUCTION

It has been increasingly recognized that tumor stromal components, including cellular components, cytokines or growth factors, extracellular matrix play major roles in tumor metastases which cause more than 90% cancer mortality [1]. Although several large-scale studies have described the extensive molecular changes in tumor-associated stromal cells during tumor progression that may contribute to metastasis, the critical factors promote the formation of pro-metastatic niches and boost the vicious tumor-stroma interactions remaining largely unknown.

The complex interactions among tumor cells, stromal cells, and extracellular matrix compositions are indispensably mediated through networks of cytokines or growth factors, which have been implicated in tumor cell growth [2], migration and invasion [3], angiogenesis [4], host immune response [5], as well as maintenance of cancer stem cells [6], through their interaction with specific receptors. Although much effort have been focused on identifying key cytokine receptors to facilitate developing targeted small-molecule antagonists or therapeutic antibodies, the fact is that cytokines are much more redundant than the known receptors so that many cytokines do not have matched receptors being identified [7]. In addition, many individual cytokines are themselves pleiotropic, exerting multiple actions, by activating multiple signaling pathways wherein different signaling pathways differentially contribute to different functions [8].

In this study, we therefore analyzed the expressions of a large panel of cytokines (132 known cytokines and growth factors) in several tumor-stromal, as well as normal-stromal clinical breast cohorts. The results consistently indicated the potential importance of connective tissue growth factor (CTGF) in tumor progression. In particular, CTGF is preferentially produced in tumor cells, and the elevated CTGF gene expression in tumor cells significantly correlates with poor clinical prognosis in breast tumors. Furthermore, in our tissue microarray analysis on 84 patient-derived xenograft models, high protein expression of CTGF correlates remarkably with the stroma-rich tumors that have poor clinical prognosis and outcome. For breast cancer, especially the triple-negative subtype, patients with stroma-rich tumors have shown a significant higher risk of poor prognosis and worse outcome compared to those with stroma-poor tumors [9], which neither currently used clinic-pathological parameters nor molecular profiling techniques are able to categorize this set of patients with respect to prognosis [10, 11].

CTGF has previously been identified as a fibrogenic cytokine that is highly expressed in wound healing and fibrotic lesions [12]. In human cancers, the pleiotropic functions of CTGF have been investigated, including the function as an oncoprotein in glioma [13] and melanoma [14], but a tumor-suppressor in lung cancer and colon cancer [15, 16]. In breast cancer, studies have shown that CTGF cooperates with other genes to mediate osteolytic metastasis, and high expression of CTGF mRNA in the bulk tumor correlated with advanced tumor stages [17], however, the mechanistic origin of CTGF has rare been explored. Whether the high level of CTGF is from tumor cells or stromal cells, and furthermore, whether CTGF mediates tumor-stroma dialogue and how CTGF regulates tumor progression in the microenvironment have not yet been clearly shown. Our data show that CTGF in tumor epithelial cells but not stromal cells had significant clinical relevance, and through a series of bioinformatics and biological analyses, we also identified that 1) CTGF facilitated tumor growth and metastasis via promoting the deposition and orientation of collagen I fibers at the primary tumor stroma; 2) CTGF was capable to promote tumor cell migration, invasion and mammosphere formation via inducing epithelial-mesenchymal transition (EMT); and 3) the CTGF-tumor necrosis factor receptor I (TNFR1)-IκB autocrine signaling is the predominant mechanism in CTGF-mediated tumor progression. Our data provided amble evidence that targeting the CTGF-TMFR1-IκB signaling is a promising strategy to prohibit breast tumor progression.

## RESULTS

### High expression of CTGF in breast tumor epithelial component correlates with poor clinical prognosis and outcomes

The interaction between tumor and stromal cells and their signaling output mould the microenvironment to support tumorigenesis and metastasis [18]. In this context, cytokines, chemokines, as well as growth, angiogenesis and developmental factors are important mediators for the cell-cell interactions. We first analyzed the gene expressions of 132 such proteins [19] in patient-matched normal epithelium, normal stroma, tumor epithelium and tumor-stroma specimens from 20 ductal breast carcinoma in situ, 18 invasive ductal breast carcinoma, and 28 normal breast samples (GSE14548) (Supplementary Table S1–S2, Supplementary Figure S1A–S1B). Our results showed that 9 out of the 132 cytokines or factors were significantly up-regulated in the stromal cells (fold change > 2 and *p* < 0.05), and so were 2 cytokines or factors in the epithelial tumor cells when comparing invasive ductal carcinoma with ductal carcinoma *in situ* and normal tissues. *CTGF* was found as the top highly expressed cytokine in both the epithelial tumor and stromal cells. In particular, *CTGF* had a 2.77-fold increase in epithelial tumor cells (*p* = 0.03) and 2.45-fold increase in stromal cells (*p* = 0.002). Other elevated cytokines include *INHBA* in epithelial tumor cells, and *CCL20*, *INHBA*, *TNFSF13*, *GH1*, *IL7*, *CXCL11*, *IL22*, and *IFNG* in stromal cells.

**Figure 1:**
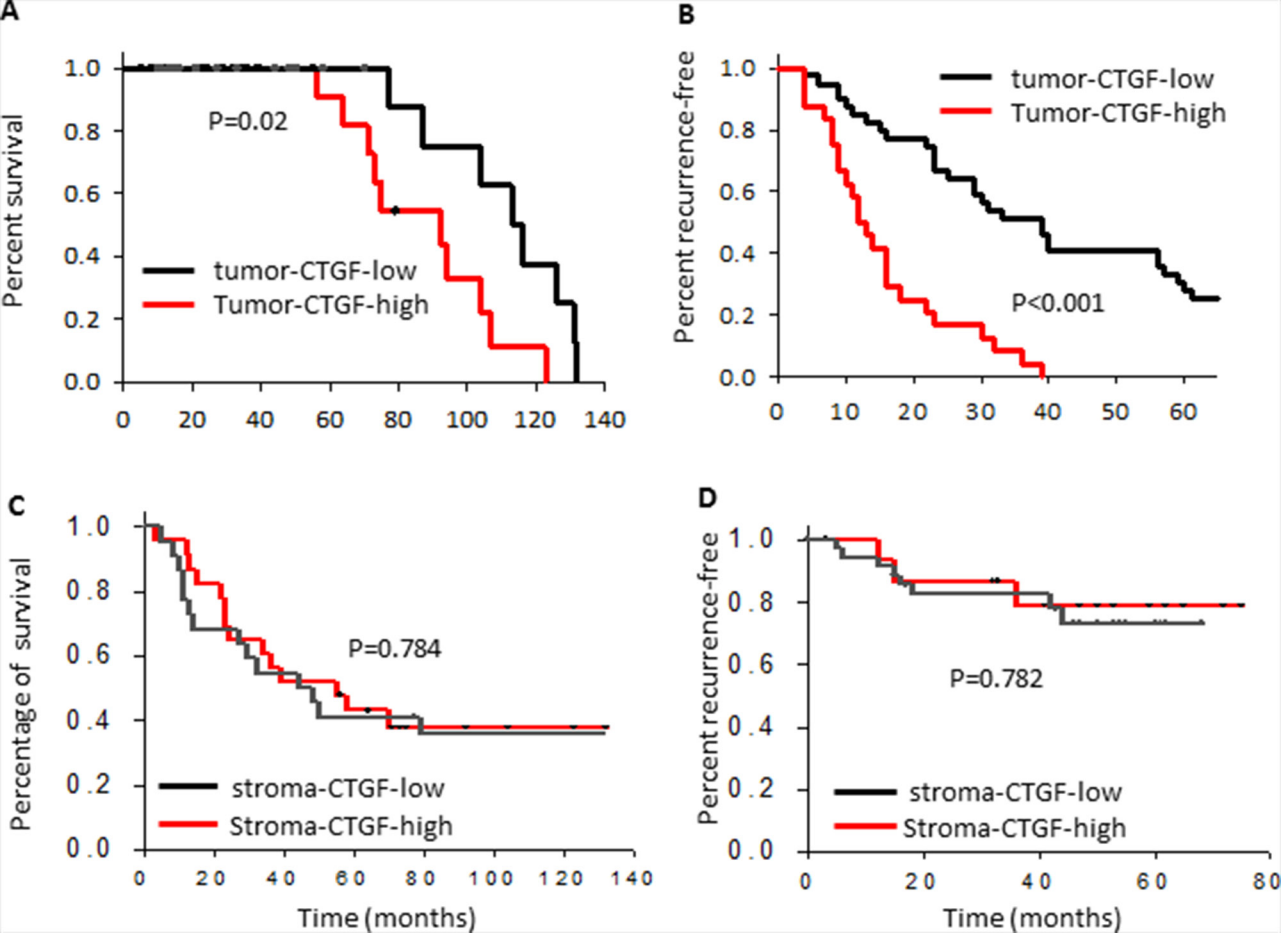
High expression of CTGF in breast tumor epithelium correlates with poor clinical prognosis and outcomes Kaplan–Meier curves showing the overall survival and recurrence-free survival of patients with low or high tumor epithelial or stroma CTGF expression in the combined cohort of 103 breast cancer patients, *P* values were determined by log rank test.

We next searched the *CTGF* expression in published breast cancer clinical datasets to see whether the high expression of *CTGF* has any clinical relevance and whether the tumor-*CTGF* and stroma-*CTGF* have similar clinical relevance. Our analysis showed that high expression of *CTGF* in the bulk tumor specimens correlated with advanced TNM stages (Supplementary Figure S1C–S1E) in a 167 breast tumor cohort (GSE4382). Furthermore, high expression of *CTGF* in basal, HER2-positive and luminal B subtype tumors correlated significantly with shorter overall survival time (Supplementary Figure S1F–S1H). However, when we analyzed the datasets with separate stroma and tumor profiling [20, 21], we found that only high tumor-*CTGF* significantly correlated with shorter overall survival (*p* = 0.02) (Figure 1A), the patient survivals from high stroma-*CTGF* group and low stroma-*CTGF* group was no difference (*p* = 0.54) (Figure 1C). Furthermore, high tumor-*CTGF* correlated with remarkable earlier recurrence (*p* < 0.001) (Figure 1B), while the patient recurrence time between high stroma-*CTGF* group and low stroma-*CTGF* group was no difference either (*p* = 0.12) (Figure 1D).

**Figure 2:**
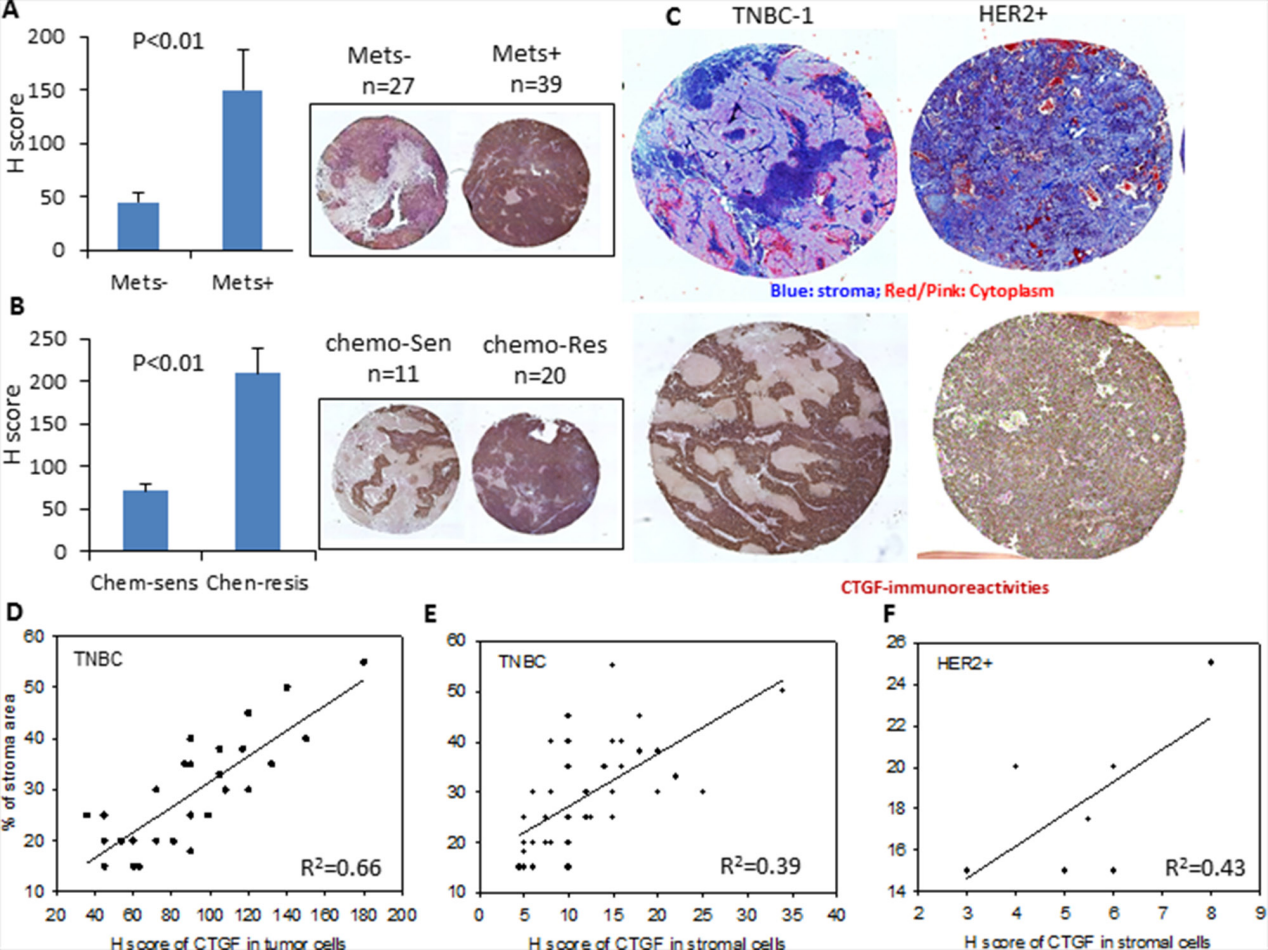
Protein expression of CTGF in PDX breast tumors **A–B.** Quantitative graphs and representative CTGF immunohistochemistry staining of the breast tumor PDX tissue microarray. H score was calculated as described in Methods. *P* values were determined by student *t*-test. **C.** Representative images showing the Masson's trichrome staining and CTGF immunohistochemistry staining of the triple-negative (TNBC) and HER2-positive (HRE2+) breast tumor PDX tissue microarray. **D.** Correlation between the H scores of CTGF in tumor cells and % of stroma area in the TNBC tissue microarrays. R^2^ = 0.66. **E.** Correlation between the H scores of CTGF in stromal cells and % of stroma area in the TNBC tissue microarrays. R^2^ = 0.39. **F.** Correlation between the H scores of CTGF in stromal cells and % of stroma area in the HER2+ tissue microarrays. R^2^ = 0.43.

In addition to the transcriptional analysis from public databases, we investigated the protein expression of CTGF in our tissue microarray set from 76 triple-negative and 8 HER2-positive patient-derived xenograft (PDX) breast tumors [22]. CTGF-immunoreactivities were examined at the cytoplasm of tumor cells and stroma rich areas (e.g., lymphatic vessels, blood vessels, fibroblasts, and extracellular stroma areas seen in Masson's trichrome staining, Supplementary Figure S1I). While CTGF expressed mildly at the stroma areas, its expression in the tumor cells was much intense. In particular, the CTGF expression at tumor areas was ~3.3-fold higher in the primary tumors with metastasis than those in the primary tumors without metastasis, and ~3-fold higher in the chemo-resistant tumors than those in the chemo-sensitive tumors (Figure 2A–2C). Furthermore, we found that high protein expression of CTGF in the tumor epithelial component correlated well with the enlarged stroma areas in the triple-negative tumors (R^2^ = 0.66), but the percentage of stroma cells expressing CTGF seemed not have such relation (R^2^ = 0.39) (Figure 2D, 2E). Stroma-rich breast tumors have been reported to correlate with poor clinical prognosis and outcome, especially in the triple-negative subtype [9]. In the PDX tumor tissue microarray, we examined in general less stroma in the 8 HER2-positive tumors than those in the triple-negative tumors, but the CTGF expression in the stroma cells of HER2-positive tumors didn't correlate with the stroma area either (Figure 2F). These results reinforce the potential significance of tumor-derived CTGF in tumor progression.

### Expression of CTGF highly correlates to EMT markers

**Figure 3:**
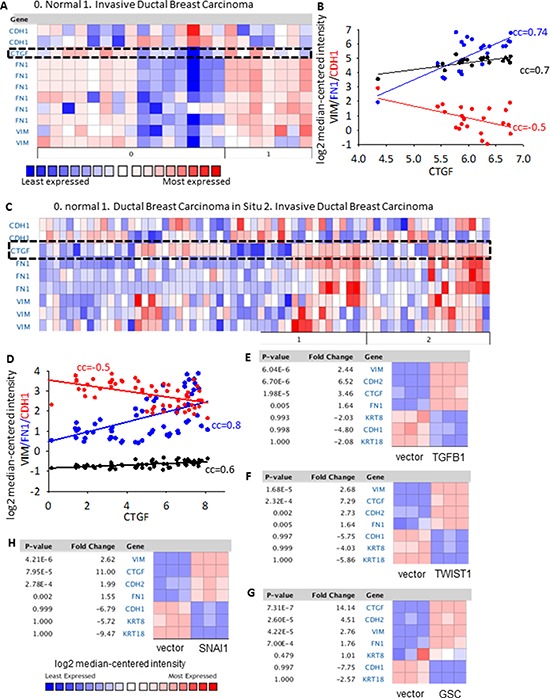
Co-expression of CTGF with EMT markers in clinical breast tumor cohorts and breast cancer cell lines **A–B.** The co-expression analysis between CTGF and *FN1, VIM, CDH1* in the *n* = 22 clinical breast tumor cohort [24]. All gene probes were listed for *FN1, VIM* and *CDH1*. The correlation co-efficient (cc) were calculated based on the log2 median-centered intensity of *FN1, VIM, CDH1* and *CTGF.* **C–D.** The co-expression analysis between CTGF and *FN1, VIM, CDH1* in the *n* = 66 clinical breast tumor cohort [25]. **E–H.** CTGF gene expression in a series EMT-induced human mammary epithelial cell (HMLER) cell lines, including over-expression of EMT transcription factor Goosecoid (GSC), Snail, or Twist, and induction of EMT by TGFB1 transfection. *P* values were determined by student *t*-test. Fold change was log 2 scale.

EMT is a fundamental process in organ fibrosis and has been demonstrated to be closely related to cancer progression [23]. To see whether CTGF has any connection with EMT, we first performed a co-expression analysis between CTGF and EMT markers in clinical breast tumors and a series of cell lines. From two independent clinical breast cohorts (*n* = 22 [24] and *n* = 66 [25] respectively), we examined that the increased expression of *CTGF* in invasive ductal breast tumors positively correlated with the increased expressions of mesenchymal markers fibronectin (*FN1*) and vimentin (*VIM*), while negatively correlated with the expression of epithelial marker E-cadherin (*CDH1*) (Figure 3A–3D). From a series of EMT-induced human mammary epithelial cell (HMLE) cell lines, including over-expression of EMT transcription factor Goosecoid, Snail, or Twist, and induction of EMT by TGF-β (GSE24202), *CTGF* showed a consistent elevation in all the EMT-induced cell lines parallel to the increased mesenchymal markers *CDH2*, *FN1*, *VIM* and decreased epithelial markers *CDH1*, *KRT18*, *KRT8* (Figure 3E–3H), although overlapping and unique contributions of each inducer to the EMT program are not the same [26]. Furthermore, the correlation between CTGF and EMT was manifested in basal, luminal A and luminal B subtypes rather than HER2-positive subtype breast tumors (Supplementary Figure S1J). In tumor-adjacent normal breast tissues, the expressions of *CTGF* were also examined to positively correlate with *FN1* and *VIM* and negatively correlate with *CDH1* (Figure 3A, 3C). These results indicate a strong correlation between CTGF and EMT in breast tumors.

### CTGF promotes tumor cell migration, invasion, and mammosphere formation via EMT

**Figure 4:**
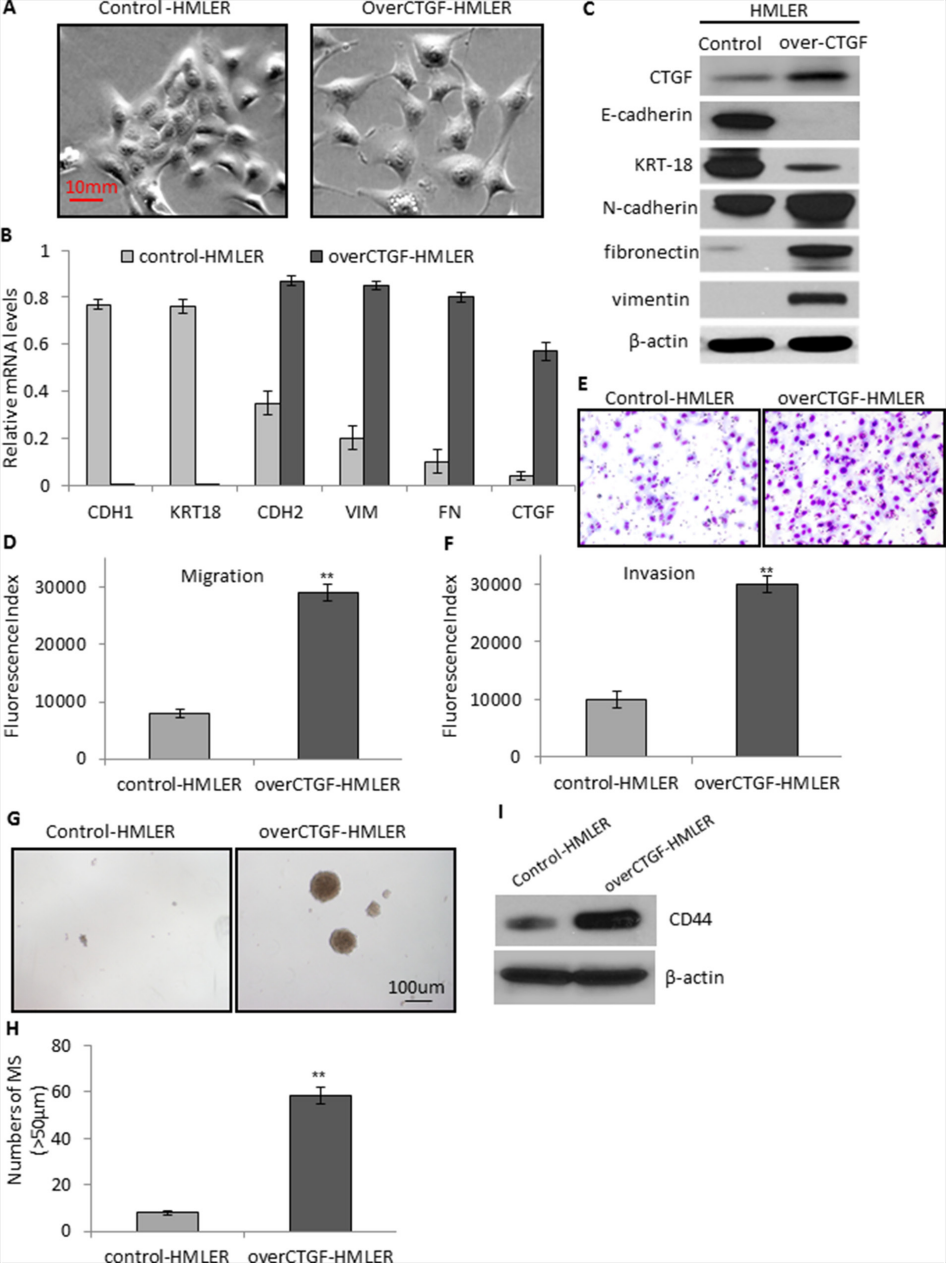
Enforced expression of CTGF promotes tumor cell migration, invasion, and mammosphere formation via EMT **A.** Representative phase-contrast images showing the elongated morphology of over-CTGF-HMLER cells which is typically associated with mesenchymal phenotype. **B.** RT-PCR analysis of the EMT genes and CTGF in the control and over-CTGF-HMLER cells. Relative gene expression level to GAPDH was plotted. **C.** Western blot analysis of the EMT markers and CTGF in the control and over-CTGF-HMLER cells. **D–E.** Quantitative graphs and representative membrane images showing the migration ability of the control and over-CTGF-HMLER cells measured by the Boyden Chamber assay. **F.** Quantitative graphs showing the invasion ability of the control and over-CTGF-HMLER cells measured by coating Matrigel in the Boyden Chamber assay. **G–H.** Enforced expression of CTGF in the HMLER cells promoted the number and size of mammospheres. **I.** Western blot analysis of enriched CD44 expression in the mammospheres. **P < 0.01, *vs* control. *P* values were determined by student *t*-test.

To clarify whether CTGF is an independent inducer for EMT or its elevation during EMT is simply a secondary phenomenon, we performed functional studies on breast cancer cell lines. Whereas many individual genetic mutations have been cataloged in numerous breast cancers, none is involved universally in all human tumors and cell lines. In this context, we selected the HMLER and HMLER-snail [27] cell lines as models to enforce the expression of CTGF or deplete the expression of CTGF, respectively. The HMLER cells are derived from normal human mammary epithelial cells through introducing 3 oncogenes whose regulatory pathways are commonly altered in naturally arising tumors [28]. HMLER cell line has a very low CTGF expression, and HMLER-snail cell line has the highest CTGF expression among all the EMT-induced HMLER cell lines (Supplementary Figure S2A). We established a stable HMLER cell line expressing CTGF through retroviral induction (designated as over-CTGF-HMLER). We found that although the HMLER cells expressing a control vector retained an epithelial morphology with tight cell-to-cell adhesion, over-CTGF-HMLER cells displayed an elongated morphology typically associated with mesenchymal phenotype (Figure 4A). Further, this morphologic change in over-CTGF-HMLER cells was accompanied by marked reduction of E-cadherin and KRT18 expressions and increased expressions of N-cadherin, fibronectin and vimentin at both mRNA and protein levels (Figure 4B–4C). These findings suggested that enforced expression of CTGF in HMLER cells can result in EMT induction. The over-CTGF-HMLER cells had an enhanced migration and invasion ability with about 3-fold increase than that in the control vector cells (Figure 4D–4F). Furthermore, we also found that over-CTGF-HMLER cells formed significantly more and larger number of mammospheres than the control cells (Figure 4G–4H). The formed mammosphere populations were found to be further enriched in protein levels of CTGF as well as CD44, which support a role of CTGF in driving the formation of mammospheres (Figure 4I).

**Figure 5:**
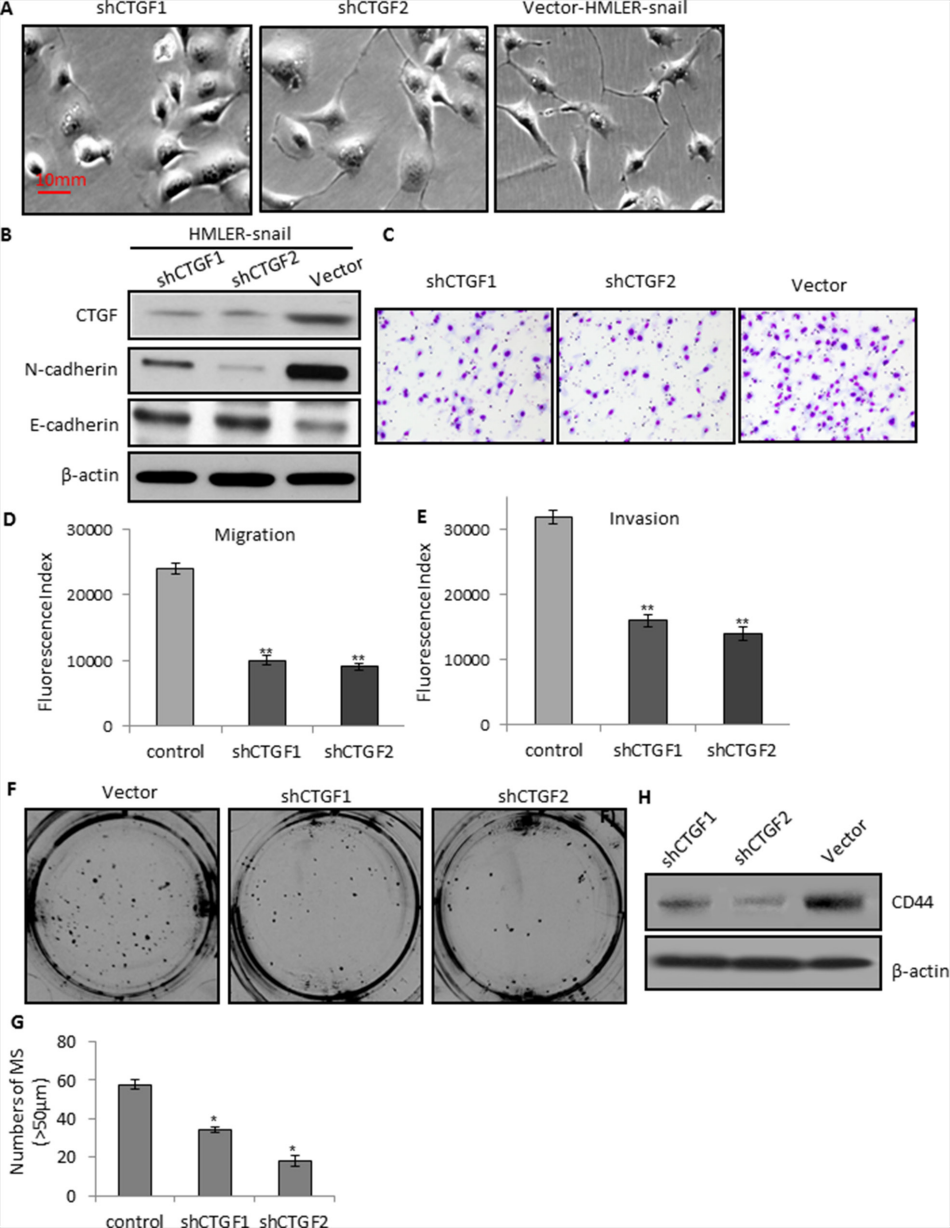
CTGF depletion reduces tumor cell migration, invasion, and mammosphere formation via MET **A.** Representative phase-contrast images showing the epithelial morphology of the shCTGFs knockdown cells. **B.** Western blot analysis of the CTGF and N-cadherin, E-cadherin in the control and shCTGFs cells. **C–D.** Quantitative graphs and representative membrane images showing the migration ability of the control and shCTGFs cells measured by the Boyden Chamber assay. **E.** Quantitative graphs showing the invasion ability of the control and shCTGFs cells measured by coating Matrigel in the Boyden Chamber assay. **F–G.** CTGF depletion in the HMLER-snail cells reduced the number and size of mammospheres. **H.** Western blot analysis of CD44 in the mammospheres. *P < 0.05, *vs* control; **P < 0.01, *vs* control. *P* values were determined by student *t*-test.

We also established two stable CTGF-knockdown cell lines by infecting HMLER-snail cells with independent CTGF shRNAs (shCTGF1 and shCTGF2). Notably, CTGF ablation in both cell lines induced a change from spindle-like mesenchymal morphology of HMLER-snail cells into epithelial morphology by manifesting an increased cell-to-cell adhesion (Figure 5A). Consistent with the phenotypic change associated with CTGF-depletion was an increased expression of epithelial marker E-cadherin (Figure 5B). Thus, CTGF depletion resulted in a reversal of EMT in HMLER-snail cells. Furthermore, CTGF depletion also markedly reduced the migration and invasion abilities of HMLER-snail cells in both of the CTGF ablation cell lines (Figure 5C–5E). This reduction of invasion should not be due to reduced cell proliferation, as CTGF ablation did not change the proliferation rate of HMLER-snail cells (Supplementary Figure S2B). We also found that CTGF ablation in both cell lines led to significant reductions of mammosphere formation than that of the control cells (Figure 5F–5G) and CD44 expression decreased in shCTGF1 and shCTGF2 cells (Figure 5H). Collectively, these findings suggest that CTGF is able to induce EMT and stem cell-like characteristics associated with aggressive phenotype of breast cancers.

### CTGF facilitates tumor growth and metastasis via promoting the deposition and orientation of collagen I fibers

**Figure 6:**
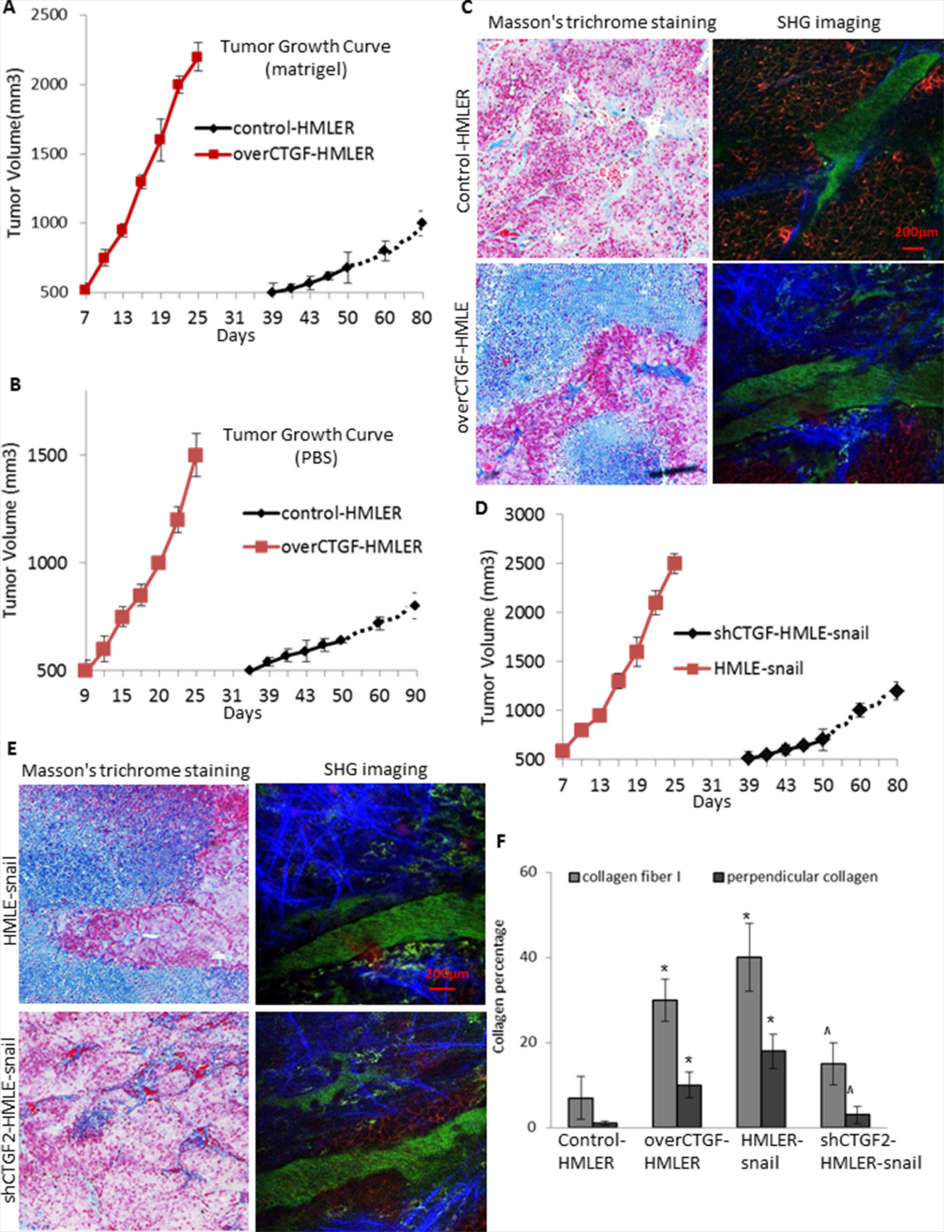
CTGF facilitates tumor growth *in vivo* via promoting the deposition and orientation of collagen I fibers **A–B.** *In vivo* tumor growth of the over-CTGF-HMLER cells and control HMLER cells mixed with Matrigel (A) and PBS (B) **C.** Representative images showing the Masson's trichrome staining of the tumor connective tissue, and second harmonic generation (SHG) imaging of the intra-tumoral collagen I fibers, of the Matrigel-mixed over-CTGF-HMLER tumors. **D–E.** *In vivo* tumor growth of the shCTGF-HMLER cells (D), and representative Masson's trichrome staining and SHG imaging of the intra-tumoral collagen I fibers. **F.** Quantification of the percentage of collagen fiber and perpendicular collagen fiber to the overall connective tissue area. Mouse tumor sections (10 μm thick) were cut serially, and one section from every 100 μm was stained by Masson's trichrome staining, and the adjacent section was imaged by SHG imaging. In each section 5–10 fields were quantified. *N* = 4 mouse tumors in each group. *P < 0.05, *vs* control HMLER; ^ *P* < 0.05, *vs* HMLER-snail. *P* values were determined by student *t*-test.

We further performed animal experiments to examine the function of CTGF in tumor development *in vivo*. 2 × 10^6^ over-CTGF-HMLER or control-HMLER cells were mixed with equal volumes of PBS or Matrigel prior to orthotopic injection. Although both over-CTGF-HMLER and control-HMLER cells mixed with Matrigel formed tumors in all injected mice, over-CTGF-HMLER cells with Matrigel led to 32 days earlier on tumor formation and much more rapid tumor growths than those in the control cell-injected mice (Figure 6A). Further, concomitant lung metastases were observed in 73% (11/15) of the mice bearing over-CTGF-HMLER tumors while no metastasis (0/15) detected when an equal number of control cells were injected into mice (*p* < 0.001, Fisher Exact Test). Brain metastases were also observed in one mouse bearing over-CTGF-HMLER tumor (Supplementary Figure S2C–S2E). Control-HMLER cells mixed with PBS but not Matrigel formed tumors in 5/15 (33%) mice, and over-CTGF-HMLER cells mixed with PBS elicited tumor formation in 15/15 (100%) injected mice (*p* = 0.012, Fisher Exact Test), with 25 days earlier on average tumor formation than the control cell-injected mice (Figure 6B). These results suggest that ectopic expression of CTGF is sufficient to induce EMT and subsequent cancer stem cell-like properties, including tumor initiation and metastatic competence in transformed human mammary epithelial cells.

Matrigel has been shown to affect the efficiency or latency of tumor formation because it contains abundant extra-cellular matrix proteins [28]. Since over-CTGF-HMLER cells alone without Matrigel elicited tumor formation in 100% (15/15) of injected mice while the control-HMLER cells only led to 33% tumor take rate, we reasoned that the over production of CTGF by the tumor cells might create a more physiologic microenvironment that would contribute positively to tumor formation of the HMLER cells. To test this notion, we performed the Masson's trichrome staining on the connective tissue of the tumors (Figure 6C, 6F). Comparing to the control-HMLER tumors, the over-CTGF-HMLER tumors contained averagely 20% more connective tissue. Collagen I fibers form a key component of the extra-cellular matrix in breast cancers [29]. When we further quantified the intra-tumoral collagen I fibers by second harmonic generation (SHG) imaging using two-photon laser scanning microscope, the results showed that not only the fibrous areas were enlarged in the over-CTGF-HMLER tumors, the average intensity of each fiber was increased which may due to the fibrotic self-assembly, and further, we detected ~30% of the fibers aligned perpendicular to the over-CTGF-HMLER tumor edges, which provided a path for the tumor cells to follow and encouraged spreading [29]. In contrast, in the shCTGF2-cell formed tumors, we found less fibrous areas and less perpendicular aligned fibers to tumor edge, accompanying with reduced tumor growths (Figure 6D–6F). All these findings suggest that CTGF is not only an important mediator of EMT for tumor cells but also a potent activator for breast tumor stroma to support tumor growth and metastasis.

### CTGF activates the TNFR1-IκB signaling in mediating EMT

Although it was called CTGF when it was discovered [30], CTGF does not behave like a traditional growth factor or cytokine since it does not appear to have a unique receptor to which it binds with high affinity to induce signal transduction. It may be more accurate to consider CTGF as a matricellular protein that modulates the interaction of cells with the matrix to modify the cellular phenotype and cellular functions through multiple signaling pathways depending on the cell type and context [31].

**Figure 7:**
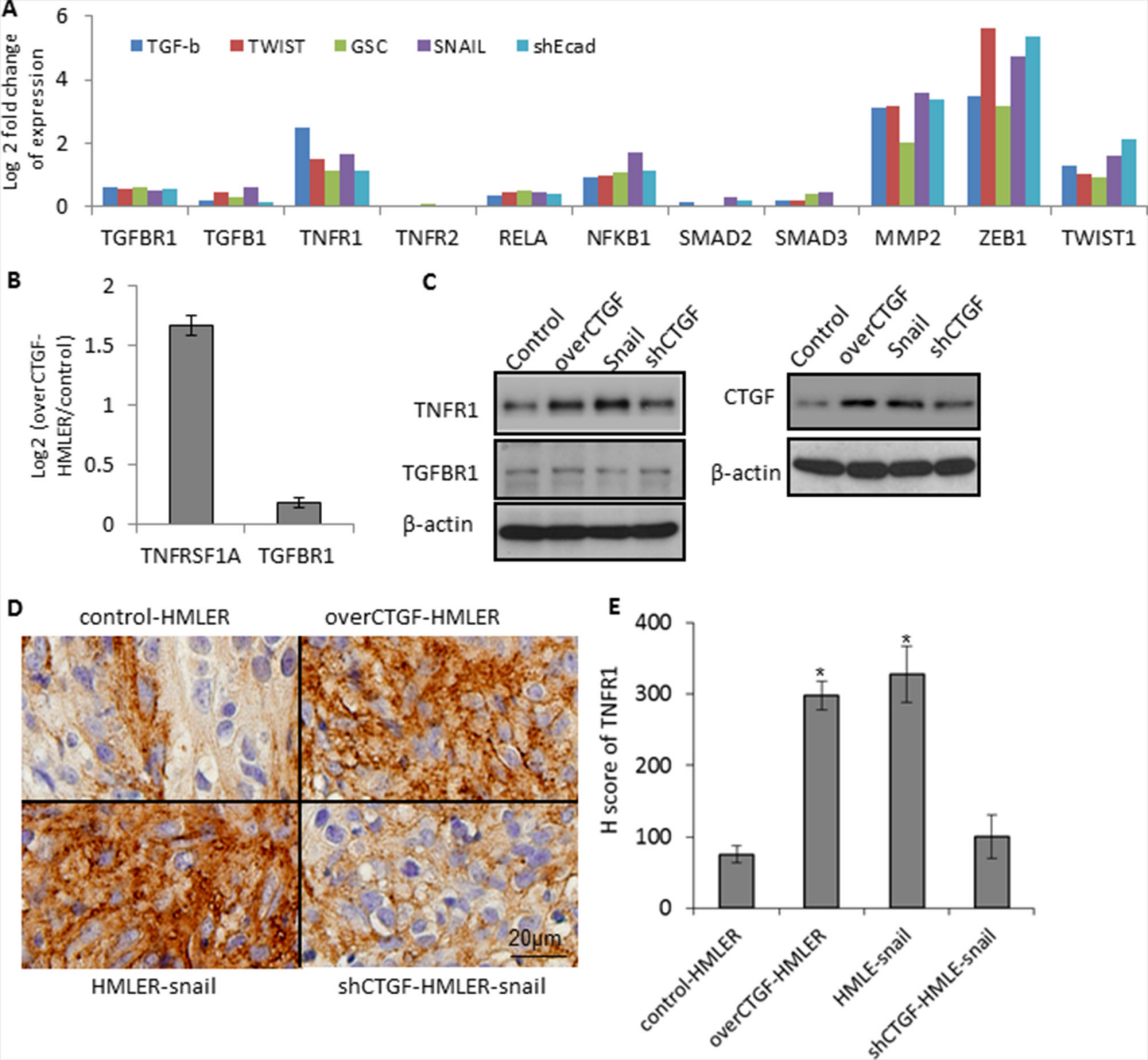
Up-regulation of TNFR1-IκB signaling in the CTGF-high tumor cells **A.** Gene expressions of TNFα and TGF-β pathways in a series HMLER cell lines, including over-expression of GSC, Snail, or Twist, and induction of EMT by TGF-β, and knocking-down E-cadherin by shEcad. Log 2 fold change of gene expression was plotted. **B–C.** RT-PCR and Western blot analyses of the TNFRSF1A and TGFBR1 in the indicated cell lines. **D–E.** *In vivo* protein expression of TNFR1 in the indicated xenograft tumors. H score was calculated as described in Methods. *N* = 4 mouse tumors in each group. *P < 0.05, *vs* control HMLER. *P* values were determined by student *t*-test.

Considering that CTGF has been shown to interact with TNF and TGFβ signalings while no other known CTGF-specific receptors [32, 33], we first focused on examining the gene expression changes of these pathways. Microarray profiling of the control HMLER and over-CTGF-HMLER cells revealed that genes in the TNFR1 pathway (*TNFR1*, *RELA*, *NFκB1*) were up-regulated (log_2_ (fold change) > 1.5, *p* < 0.01), but not *TNFR2* in the over-CTGF-HMLER cells. In addition, expressions of *MMP2*, *ZEB1* and *TWIST1* in the downstream of TNF-α pathway increased significantly (log_2_ (fold change)> 1, *p* < 0.05). However, *TGF-β*, *TGF-βR1* and some molecules in the TGF-β pathway (*SMAD2*, *SMAD3*) did not change (Figure 7A). *TNFR1* expressions in the series of EMT-induced HMLER cell lines were also consistently up-regulated, and they all have high expression of *CTGF* (Figure 7B). Protein expressions of TNFR1 in the various cancer cell lines were in concord with the gene expressions of *TNFR1* and tumor immunostaining of TNFR1 further confirmed that TNFR1 expressed higher in the over-CTGF-HMLER and HMLER-snail tumors than that in the control-HMLER and shCTGF2 tumors (Figure 7C–7E).

**Figure 8:**
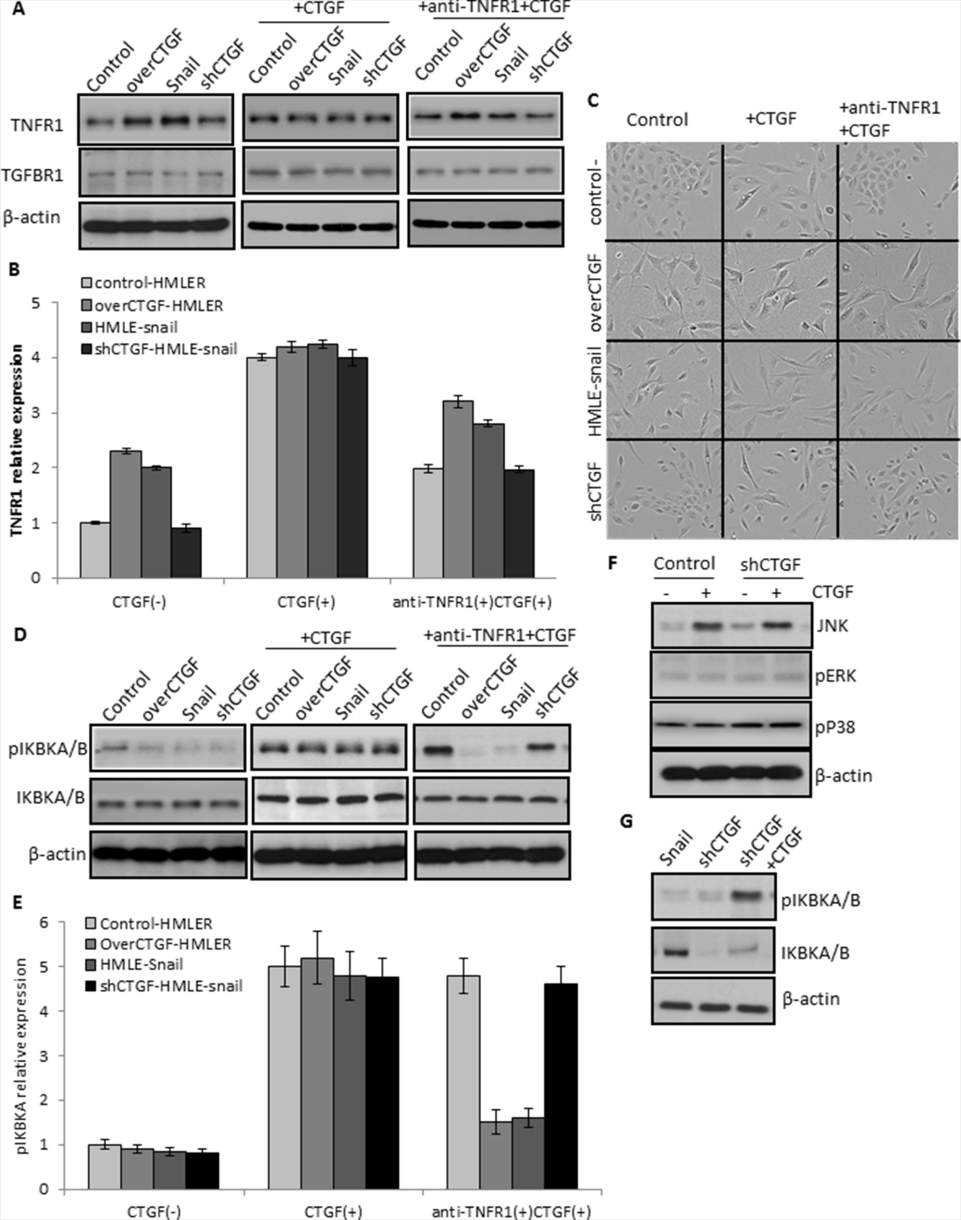
Essential role of TNFR1 in mediating the function of CTGF signaling **A–B.** A precedent treatment with anti-TNFR1 monoclonal antibody (10 μg/ml) more profoundly prevented the increase of CTGF-induced TNFR1 expression in the CTGF-highly expressed cells (over-CTGF-HMLER and HMLER-snail) than that in the CTGF-lowly expressed cells (control-HMLER and shCTGF2). **C.** Representative phase-contrast images showing the cell morphology changes in the indicated cell lines and treatments. **D–E.** The activation of IKBKA and IKBKB by CTGF could be specifically attenuated by the anti-TNFR1 antibody in the CTGF-high tumor cells. **F.** JUK but not ERK1/2 and p38 in the MAPK signaling pathway was up-regulated by exogenous CTGF in the CTGF-low tumor cells. **G.** Knockdown CTGF in the HMLER-snail cells down-regulated the IKBKA and IKBKB, and such down-regulation could be rescued by exogenous CTGF. All experimental data are representative of at least two independent experiments performed in triplicate. Data are expressed as mean ± s.e.m.

To confirm the role of TNFR1 in mediating the function of CTGF, we used the TNFR1 specific antagonist antibody and recombinant human CTGF to treat tumor cells. Although the recombinant human CTGF (100 ng/ml) caused the increase of TNFR1 expression in all the treated cancer cells, the precedent treatment with anti-TNFR1 monoclonal antibody (10 μg/ml) more profoundly prevented the increase of CTGF-induced TNFR1 expression in the CTGF-highly expressed cells (over-CTGF-HMLER and HMLER-snail) than that in the CTGF-lowly expressed cells (control-HMLER and shCTGF2) (Figure 8A–8B). These results showing the more sensitive responses of the TNFR1 expression to TNFR1 blockage and CTGF addition implicated the possible CTGF-TNFR1 interaction. In parallel, cells under the different treatments appeared morphological changes pertinent to EMT or MET, for example, the morphologies of control-HMLER and shCTGF2 cells changed from cobblestone-like epithelial appearance to an elongated, spindle-like fibroblastic shape after treated with recombinant human CTGF, and changed back to epithelial appearance once anti-TNFR1 was treated for 3 days (Figure 8C).

TNFR1 signaling induces activation of many genes, primarily controlled by two distinct pathways, NF-κB pathway and the MAPK cascade. To further support that TNFR1 mediated the function of CTGF in inducing EMT, we detected the downstream signaling molecules of TNFR1 in the tumor cells. The results showed that the activation of IKBKA and IKBKB by CTGF could be specifically attenuated by the anti-TNFR1 antibody in the CTGF-high tumor cells (Figure 8D–8E). JUK but not ERK1/2 and p38 was up-regulated by exogenous CTGF in the CTGF-low tumor cells (Figure 8F). Further, knockdown CTGF in the HMLER-snail cells down-regulated the IKBKA and IKBKB, and such down-regulation was rescued by exogenous CTGF (Figure 8G). Altogether, these results indicated that the enhanced CTGF could activate the TNFR1-IκB signaling of tumor cells in mediating EMT in transformed human mammary epithelial cells.

### Drug treatments target CTGF-TNFR1-IκB signaling

**Figure 9:**
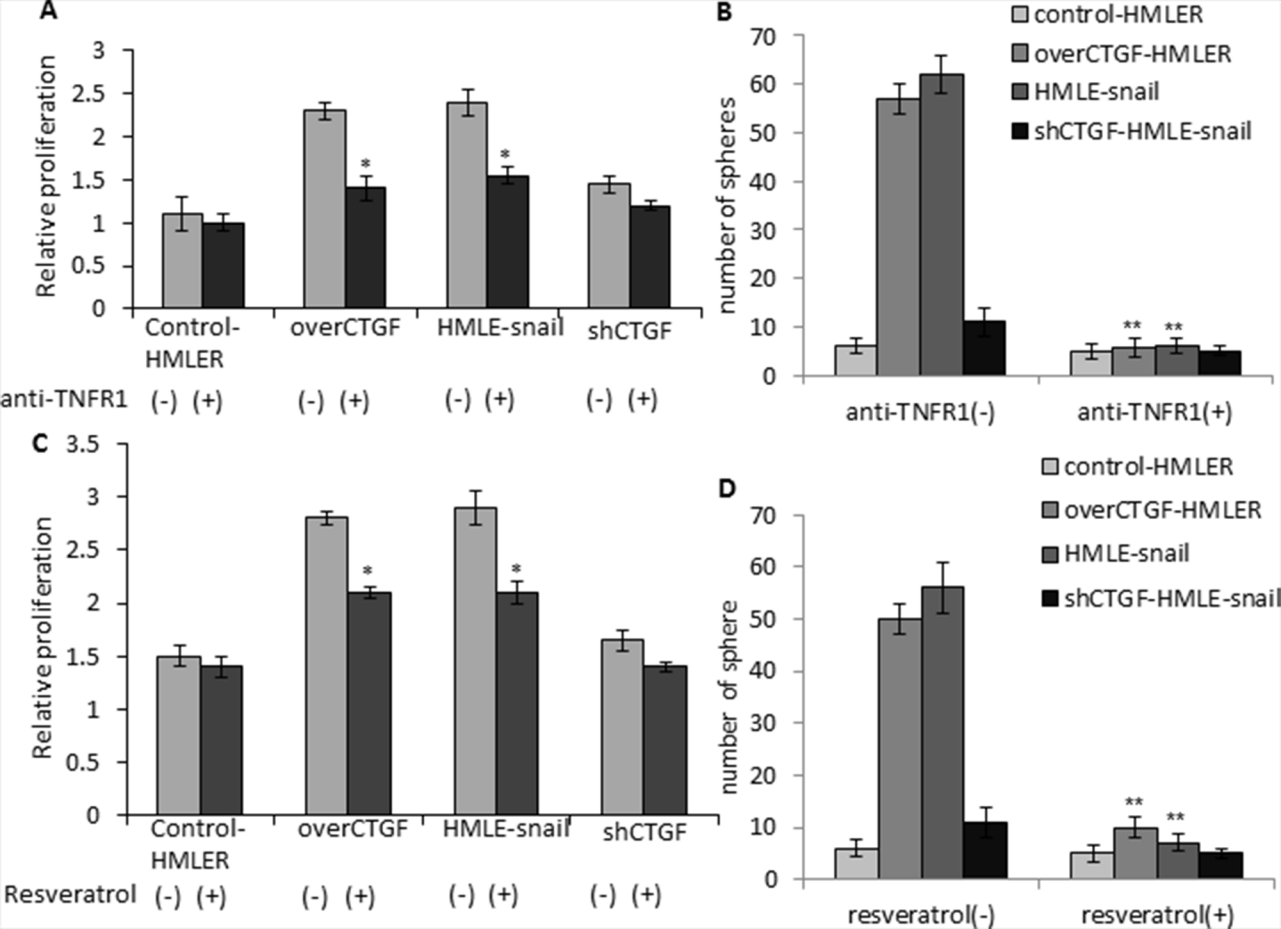
Anti-TNFR1 and resveratrol specifically inhibit the cell growth and mammosphere formation of the CTGF-high tumor cells **A–B.** Cell proliferation and mammosphere formation were significantly inhibited by the anti-TNFR1 treatment in over-CTGF-HMLER and HMLE-snail cells but not control-HMLER cells or shCTGF-HMLE-snail. **C–D.** Cell proliferation and mammosphere formation were significantly inhibited by resveratrol treatment in over-CTGF-HMLER and HMLE-snail cells but not control-HMLER cells or shCTGF-HMLE-snail. *P < 0.05, **P < 0.01, *vs* anti-TNFR1(−) or resveratrol (−) group of the corresponding cell lines. *P* values were determined by student *t*-test.

Since we found that CTGF is elevated in invasive breast tumors, induces EMT, facilitates tumor growth and metastasis via remodeling collagen I fibers, we first attempted to find known drugs to inhibit the production of CTGF. An oral anti-CTGF compound PBI-4050 (ProMetic Life Sciences Inc.), has been shown to reduce fibrosis in multiple organs and reduce hepatocellular carcinoma in animals (http://www.prometic.com/en/therapeutics/scientific-library.php). A CTGF-specific antibody FG-3019 (FibroGen Inc.) inhibits pancreatic tumor growth and metastasis in nude mice [34], and a phase II clinical trial using FG-3019 in patients with pancreas cancer has announced positive activity (http://www.fibrogen.com/press/release/pr_1401519665). However, due to the inaccessible of these drugs, we used TNFR1 neutralizing antibody and tested whether anti-TNFR1 could suppress the stem-like and metastatic properties of CTGF-expressing cells. Anti-TNFR1 treatment was found to specifically inhibit the cell growth of over-CTGF-HMLER and HMLE-snail but not control-HMLER control cells or shCTGF-HMLE-snail in monolayer cultures (Figure 9A). The treatment of over-CTGF-HMLER cells with anti-TNFR1 was found to significantly decrease mammosphere formation by >10-fold (Figure 9B), as compared to vehicle (DMSO) treated cells. In addition, cells expressing high endogenous CTGF (HMLE-snail) also display increased sensitivity to anti-TNFR1 as evidenced by both MTT assay and reduction in mammosphere formation (Figure 9A, 9B).

Lastly, we tested the effects of an existing IκB inhibitor on the stem-like and metastatic properties of CTGF-expressing cells. In this context, we chose a natural compound from red wine, resveratrol, which has strong effects on blocking signal-dependent phosphorylation and degradation of IκB molecules to decrease NF-κB DNA binding activity [35]. Resveratrol has also been reported to attenuate CTGF up-regulation in myocardial infarction animal models and preserve contractile reserve [36]. The results showed that resveratrol treatment (200 μmol/l) inhibited the cell growth of over-CTGF-HMLER remarkably but had moderate inhibition on the control-HMLER cells (Figure 9C). The treatment of over-CTGF-HMLER and HMLER-snail cells with resveratrol were found to significantly decrease mammosphere formation by >5-fold and >8-fold, respectively (Figure 9D). Taken together, these results indicate CTGF-expressing tumor cells are sensitive to TNFR1, IκB and CTGF-targeted therapies and suggest resveratrol may be effective means of targeting CTGF-expressing cell populations to impede the EMT-inducing CSC-like and metastatic phenotypes.

### DISCUSSIONS

To the best of our knowledge, this study is the first attempt to systematically compare the expressions of 132 known cytokines and growth factors in epithelium and stroma specimens from normal breast, ductal breast carcinoma in situ and invasive ductal breast carcinoma. Beyond our expectation, not many but only 10 cytokines and growth factors were identified as significantly altered ones in either tumor epithelial cells or stroma cells, and all of them were up-regulated in the invasive ductal carcinoma compared with those in the ductal carcinoma in situ and normal tissues. *CTGF* and *INHBA* (a member of the transforming growth factor β (TGF-β) superfamily) are the ones elevated in both tumor epithelial and stroma. We characterized the clinical relevance of CTGF in breast tumor progression, and the results revealed that high expression of CTGF in tumor cells but not in stroma cells significantly correlated with poor clinical prognosis and outcomes in breast tumors. Although CTGF has been implicated in fibrosis, wound repair, angiogenesis and EMT in fibroblastic cells [37], our study provides evidence that CTGF may promote metastasis by acting directly on tumor cell EMT. The EMT program has been linked to the generation of breast cancer stem cells [27] and been well documented in promoting an invasive and metastatic phenotype [38, 39]. Depletion of CTGF by RNA interference reversed EMT phenotype in well-established breast cancer mesenchymal cells. Furthermore, drug treatments that targeting the tumor CTGF-TNFR1-IκB signaling resulted in phenotypic reversal of EMT and reduced the stem cell-like property as well as cell migration and invasion. These data clearly demonstrate the distinct function of CTGF in the tumor epithelial component.

**Figure 10:**
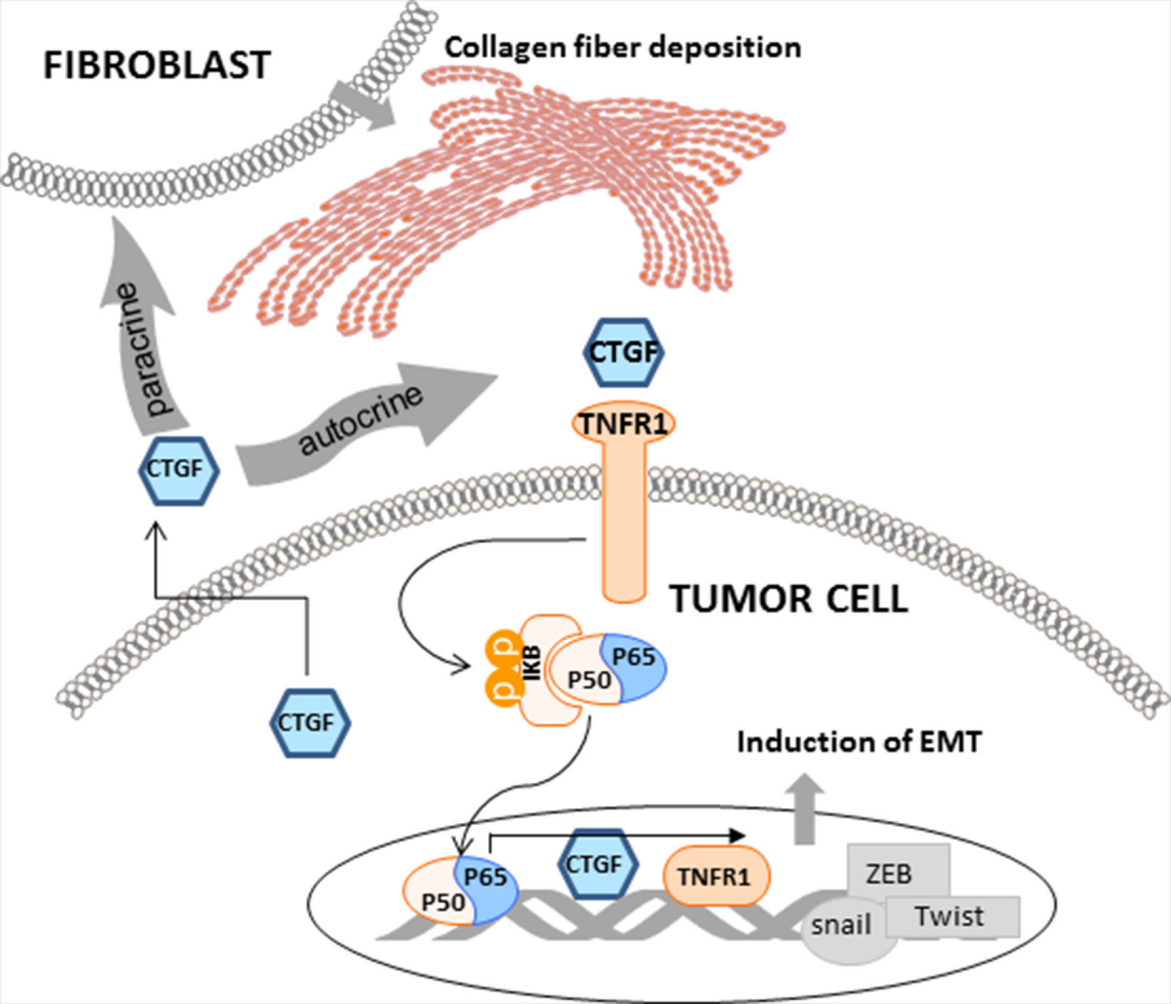
Schematic representation of the function of CTGF and associated mechanisms in tumor epithelial and stroma components CTGF is mainly derived from epithelial tumor cells. Through the CTGF-TNFR1-IκB autocrine signaling, CTGF promotes tumor EMT which contributes to the stemness and metastasis. Through promoting the deposition and orientation of collagen I fibers in the primary tumor stroma, CTGF promotes stroma-tumor interaction which facilitates tumor progression.

CTGF belongs to the CCN family, which consists of six members that all possess an N-terminal signal peptide identifying them as secreted proteins. However, no any high affinity binding receptors for CTGF has been identified, and previous studies suggest that the mechanism of CTGF in modulating the interaction of cells is largely cell type and context dependent [40]. CTGF has been reported as an adhesion factor in interacting with αvβ3 integrin to activate of ERK1/2 signal cascade in MCF-7 and MDA-MB231 breast cancer cells [41]. In our study, HMLER cells were studied, and we did not detect obvious changes of αvβ3 integrin and ERK1/2 at both gene and protein levels, suggesting the cell type specificity of CTGF signaling. Instead, we found that TNFR1 signaling was activated in the over-CTGF-HMLER cells and the specific TNFR1 antagonist antibody could attenuate the CTGF-mediated cell functions. Although no any clue indicates the physical binding of CTGF and other CCN family proteins with TNFR1, and our study didn't provide direct evidence on this either, the functional relevance of the identified CTGF-TNFR1 signaling to tumor EMT seems overt and exceptional. Upon activating TNFR1 on the tumor cells, a phosphorylation of IκB-α on Ser32 and 36 was verified in our study, and such phosphorylation leads to IκB-α degradation, which allows the p50/p65 dimer to enter the nucleus and activate Snail and Zeb2 expression to represse the E-cadherin promoter [42]. Furthermore, using the JASPAR database, we located two high scoring NF-κB sites in the human CTGF promoter at − 93/ − 86bp and − 2104/ − 2113bp, suggesting regulation by NF-κB, and p65 overexpression has been shown to increase CTGF promoter activity [43]. Taken together, these data prompt that the CTGF-TNFR1-IκB signaling activation may lead to two consequences in the tumor cells, one is the induction of EMT, and the other is the sustained autocrine of CTGF, and both of them contribute to the tumor progression (Figure 10).

In our study, CTGF was also examined to be highly deposited in stromal area in clinical breast tumors and PDX tumors, and the xenograft tumor growth and metastasis were partially facilitated by CTGF on promoting the deposition and orientation of collagen I fibers at the primary tumor stroma. Previous studies on cDNA microarray analysis identified CTGF as a candidate gene expressed in prostate stromal cells responsible for the tumor-promoting activity [44]. CTGF is a major factor in regulating the proliferation and differentiation of fibroblasts to produce mainly collagens but also many extracellular matrix (ECM) proteins [45]. The collagen fiber alignment, as well as many ECM proteins, has been defined as important factors in interacting with tumor cells to enhance tumorigenesis and metastasis [46]. In this context, we postulate that the abnormal secretion of CTGF by tumor cells is also a culprit in mediating the vicious tumor-stroma interactions. Indeed, on the one hand, the CTGF expression could be contingently augmented at the transcriptional level by the positive feedback of CTGF-TNFR1-IκB autocrine mechanism. On the other hand, we found a significant CTGF DNA copy number gain in high-grade breast tumors in a 1,992-sample analysis (Supplementary Figure S3) [47], which might be the origin of elevated mRNA and protein expressions of CTGF, as no common mutation was detected in the CTGF promoter region in breast tumors in the COSMIC database (http://cancer.sanger.ac.uk/cancergenome/projects/cosmic/). All in all, reciprocal tumor-stroma interactions could combine to produce large amount of CTGF, and the diplex functions of CTGF result in tumor progression.

Finally, we performed drug treatment studies to show the therapeutic potential of targeting the CTGF-TNFR1-IκB signaling in breast cancer models. Although the treatments were on *in vitro* cells, cell phenotypic and functional changes were confirmed to associate with reversed EMT, reduced tumor growth and metastasis. Targeting CTGF by two known drugs showed promising results in several cancer models. In comparing with CTGF, TNFR1 has been extensively studied to mediate the function of TNF-α in promoting breast cancer growth, and blockage of TNFR1 with specific antibodies was enough to impair TNF-α signaling and biological effects [48]. Although several TNF ligands were identified as elevated in both tumor epithelial and stroma in our analysis, our data showed that pre-treatment with specific anti-TNFR1 antibody almost completely blocked the effect of synthetic CTGF in the CTGF-low control-HMLER and shCTGFs cells. Furthermore, anti-TNFR1 mono-treatment could suppress the stem-like and metastatic properties of the CTGF-expressing cells, which revealed the specificity of TNFR1 in mediating the CTGF signaling in inducing tumor cell EMT. Considering that TNF-α-TNFR1 signaling is crucial in the regulation of immune responses that occur under conditions of limited immunostimulatory capacity, such as tumor surveillance [49], we anticipate that targeting TNFR1 would bring synergic anti-tumor effects by inhibiting CTGF-mediated EMT in tumor cells and modifying TNF-mediated inflammatory responses. We also tested a phytotherapeutic supplement resveratrol, in targeting the IκB phosphorylation of the CTGF-TNFR1 signaling on the EMT properties of CTGF-expressing cells and found resveratrol was able to significantly suppress the mammosphere formation as well as selectively inhibit the growth of these cells. A system biology study in using gene signature of stem cells to guide therapeutic selection for cancer has identified resveratrol as the top one-ranked breast cancer stem cell inhibitor [50]. Although this study implies the broad mechanism of resveratrol on cancer, combining with our data, it overall supports the clinical application of resveratrol in breast oncology.

Our findings are probably not unique to breast cancer. CTGF expression in B-cell acute lymphoblastic leukemia, pancreas and gastric cancer correlates to worse prognosis [51]. High expression of CTGF is a hallmark of ileal carcinoids [52], which are well-differentiated endocrine carcinomas originating from the small intestine and proximal colon. These tumors are malignant and most patients have metastatic disease at diagnosis but lacking effective treatment. Although the mechanistic insights of CTGF in these tumors may be different, the current study may provide valuable guidance and treatment strategy targeting CTGF, TNFR1 and IκB in managing these metastatic tumors.

## MATERIALS AND METHODS

### Cell culture and *in vitro* studies

The human breast cancer cell lines HMLE-Ras and HMLE-Ras-SNAIL were kindly provided by Dr. Mani Senduri (MD Anderson Cancer Center, TX). Cell line characterization or authentication was performed by the short tandem repeat profiling and passaged in our laboratory for fewer than 6 months after receipt. Knockdown of CTGF with a validated hairpin was achieved by CTGF shRNA (Santa Cruz Biotechnology). Two independent shRNAs were used to generate two CTGF knockdown cell lines. Transfections were performed using Lipofectamine2000 (Invitrogen). Puromycin (2 μg/ml) was used to select for stable cell lines. Overexpression of full-length CTGF gene was achieved by transfection of cDNA for CTGF (OriGene Technologies), which clones the open reading frame (ORF) of this gene into the pCMV6 Entry vector. TurboFectin 8.0 (OriGene Technologies) was applied to perform transient transfection. The efficiency of the knockdown and transduction was confirmed by quantitative real-time PCR, and western immunoblotting analysis.

### Cell proliferation assay

Tumor cells plated at a density of 1.5 × 10^4^ cells per well in 96-well plates were treated with compounds or with 0.1% DMSO for 24 hours followed by the addition of the CellTiter-Glo^®^ Luminescent Cell Viability Assay reagent (Promega), and then imaged by IVIS200 system (Xenogen Corporation, Alameda, CA) under bioluminescent imaging function. An average of three kinetic bioluminescent acquisitions was obtained within 5 minutes. Regions of interest (ROI) were automatically drawn over wells and quantified with the Living Image Software version 2.50.1. Data were analyzed based on total photon flux emission (photons/s) minus the background photon flux of blank wells. The mean and standard error for each treatment (singles and combination) were calculated relative to the control:

$$\%\text{ of viable cells}=\frac{P_{T}-P_{B}}{P_{C}-P_{B}}\times 100$$

where P_C_ = photon counts of the control (mean value); P_T_ = photon counts of the treated cells (mean value); P_B_ = photon counts of the blank (mean value). Data are shown as a percentage of the vehicle-treated control cells. Three separate experiments were performed, with six replicate wells for each data point.

### Mammosphere formation assay

Tumor cells treated with compounds or with 0.1% DMSO for 24 hours were collected and re-plated at 20,000 cells per ml media for culturing mammospheres in ultralow-adherent six-well plates (2.5 mL per plate) (Corning Life Sciences, USA). Mammosphere media contains mammary epithelial growth medium (MEGM, Lonza, Walkersville, M.D.) supplemented with B27, bFGF, and EGF (final concentration 20 ng/ml bFGF and 20 ng/ml EGF. The whole well montage images were acquired by ImageExpress automatic microscope and the number of mammospheres larger than 50 μm diameter was counted manually at the end of 2-week culture.

### Quantitative RT-PCR

Total RNA from cells was obtained for quantitative real-time PCR (qRT-PCR) analysis for human GAPDH (as an internal control) and VIM, FN1 CDH2, CDH, CK18, CTGF by using TaqMan Gene Expression Assays (Applied Biosystems). The specific primers are listed in Supplementary Materials. Data were analyzed with the SDS 2.1 software (Applied Biosystems).

### Western blotting

Cell lysates and immunoblotting analysis were performed as described before [53]. The antibodies are described in Supplementary Materials. Densitometric analysis was performed using ImageJ software.

### Tissue microarray and tumor section staining

Immunohistochemical staining and Masson's trichrome staining were performed on the 84 PDX tumor microarray and tumor sections. The staining for CTGF (Abcam, catalog No. ab6992), and TNFR1 (C25C1, Rabbit mAb #3736 monoclonal antibody) on the total 84 tumor samples and scoring the IHC stains were performed by HMRI Pathology Core. An H score was calculated by multiplying the fraction of positively stained tumor (percentage) by staining intensity (0, 1+, 2+, or 3+) [54]. Intensity of immunoreactivity was scored (0 and 1+ indicates negative; 2+, indeterminate; and 3+, positive for overexpression), and the percentage of tumor staining positive was visually estimated.

### Clinical datasets analysis

Clinical breast tumor gene expression data for GSE14548, GSE4382, GSE9691 and GSE24202 were downloaded from GEO; Boersma Breast (*n* = 95), Finak Breast (*n* = 59), Karnoub Breast (*n* = 22), Ma Breast (*n* = 61) and Curtis Breast (*n* = 2136) datasets were exported from Oncomine^TM^ database. All data sets were transformed to log-2 scales. PAM50 classification of tumor subtype was analyzed in the Oncomine^TM^ database for the Curtis Breast (*n* = 2136). Patient was determined as CTGF-high or –low group when the CTGF expression value was above or below the mean value in each dataset. The survival distributions were estimated by the Kaplan-Meier method, and the significance of differences between survival rates was ascertained using the log-rank test.

### *In vivo* animal experiments

Animal procedures were conducted in accordance with the guideline of IACUC and the regulations of the Animal Research and Comparative Medicine Committee of The Methodist Hospital Research Institute. Female BALB/c nude mice (5–7 weeks old; Charles River Laboratories) were anesthetized with isoflurane/O_2_ and injected with 1 × 10^6^ viable single cells in a 1:1 mixture of PBS or Matrigel (BD Biosciences) into mammary gland 4 in a total volume of 50 μL. Primary tumor growth rates were analyzed by measuring tumor length (L) and width (W), and calculating tumor volume based on the formula πLW^2^/6. Animals were euthanized when tumor diameter reached 2cm, mammary gland tumors were collected and second harmonic generation (SHG) imaging for the collagen I fiber was conducted using two-photon laser scanning microscope (Olympus, FV1000-MPE). Images were analyzed as we described before [55]. Lung and brain tissues of the mice were immediately dissected after euthanization and bioluminescent imaging was conducted to examine metastasis. Tumor tissue sections were used for immunohistological staining with anti-CTGF, and anti-TNFR1 antibodies. Lung and brain tissue sections were stained with anti-human CD44 antibody to confirm metastasis.

### Statistical tests

All experimental data presented are representative of at least two independent experiments performed in triplicate. Data are expressed as mean ± s.e.m. For comparison of groups, we used the two-tailed *t* test. To test for correlations, we calculated the Spearman's rank correlation coefficient. To compare the survival, we used the log rank test. A level of *P* < 0.05 was regarded as statistically significant. We did all calculations with SigmaPlot statistical software (version 11.2; Systat Software Inc. Chicago, Illinois).

## SUPPLEMENTARY MATERIALS FIGURES AND TABLES







## References

[1] Khamis ZI, Sahab ZJ, Sang QX (2012). Active roles of tumor stroma in breast cancer metastasis. International journal of breast cancer.

[2] Sheu BC, Chang WC, Cheng CY, Lin HH, Chang DY, Huang SC (2008). Cytokine regulation networks in the cancer microenvironment. Frontiers in bioscience : a journal and virtual library.

[3] Muller A, Homey B, Soto H, Ge N, Catron D, Buchanan ME, McClanahan T, Murphy E, Yuan W, Wagner SN, Barrera JL, Mohar A, Verastegui E, Zlotnik A (2001). Involvement of chemokine receptors in breast cancer metastasis. Nature.

[4] Neufeld G, Kessler O (2006). Pro-angiogenic cytokines and their role in tumor angiogenesis. Cancer metastasis reviews.

[5] Lin WW, Karin M (2007). A cytokine-mediated link between innate immunity, inflammation, and cancer. The Journal of clinical investigation.

[6] Korkaya H, Liu S, Wicha MS (2011). Breast cancer stem cells, cytokine networks, and the tumor microenvironment. The Journal of clinical investigation.

[7] Proudfoot AE (2002). Chemokine receptors: multifaceted therapeutic targets. Nature reviews Immunology.

[8] Bezbradica JS, Medzhitov R (2009). Integration of cytokine and heterologous receptor signaling pathways. Nature immunology.

[9] de Kruijf EM, van Nes JG, van de Velde CJ, Putter H, Smit VT, Liefers GJ, Kuppen PJ, Tollenaar RA, Mesker WE (2011). Tumor-stroma ratio in the primary tumor is a prognostic factor in early breast cancer patients, especially in triple-negative carcinoma patients. Breast cancer research and treatment.

[10] Sotiriou C, Pusztai L (2009). Gene-expression signatures in breast cancer. The New England journal of medicine.

[11] Fan C, Oh DS, Wessels L, Weigelt B, Nuyten DS, Nobel AB, van't Veer LJ, Perou CM (2006). Concordance among gene-expression-based predictors for breast cancer. The New England journal of medicine.

[12] Moussad EE, Brigstock DR (2000). Connective tissue growth factor: what's in a name?. Molecular genetics and metabolism.

[13] Edwards LA, Woolard K, Son MJ, Li A, Lee J, Ene C, Mantey SA, Maric D, Song H, Belova G, Jensen RT, Zhang W, Fine HA (2011). Effect of brain- and tumor-derived connective tissue growth factor on glioma invasion. Journal of the National Cancer Institute.

[14] Braig S, Wallner S, Junglas B, Fuchshofer R, Bosserhoff AK (2011). CTGF is overexpressed in malignant melanoma and promotes cell invasion and migration. British journal of cancer.

[15] Chang CC, Shih JY, Jeng YM, Su JL, Lin BZ, Chen ST, Chau YP, Yang PC, Kuo ML (2004). Connective tissue growth factor and its role in lung adenocarcinoma invasion and metastasis. Journal of the National Cancer Institute.

[16] Jiang CG, Lv L, Liu FR, Wang ZN, Liu FN, Li YS, Wang CY, Zhang HY, Sun Z, Xu HM (2011). Downregulation of connective tissue growth factor inhibits the growth and invasion of gastric cancer cells and attenuates peritoneal dissemination. Molecular cancer.

[17] Xie D, Nakachi K, Wang H, Elashoff R, Koeffler HP (2001). Elevated levels of connective tissue growth factor, WISP-1, and CYR in primary breast cancers associated with more advanced features. Cancer research.

[18] Quail DF, Joyce JA (2013). Microenvironmental regulation of tumor progression and metastasis. Nature medicine.

[19] Graeber TG, Eisenberg D (2001). Bioinformatic identification of potential autocrine signaling loops in cancers from gene expression profiles. Nature genetics.

[20] Boersma BJ, Reimers M, Yi M, Ludwig JA, Luke BT, Stephens RM, Yfantis HG, Lee DH, Weinstein JN, Ambs S (2008). A stromal gene signature associated with inflammatory breast cancer. International journal of cancer Journal international du cancer.

[21] Finak G, Bertos N, Pepin F, Sadekova S, Souleimanova M, Zhao H, Chen H, Omeroglu G, Meterissian S, Omeroglu A, Hallett M, Park M (2008). Stromal gene expression predicts clinical outcome in breast cancer. Nature medicine.

[22] Zhang X, Claerhout S, Prat A, Dobrolecki LE, Petrovic I, Lai Q, Landis MD, Wiechmann L, Schiff R, Giuliano M, Wong H, Fuqua SW, Contreras A, Gutierrez C, Huang J, Mao S (2013). A renewable tissue resource of phenotypically stable, biologically and ethnically diverse, patient-derived human breast cancer xenograft models. Cancer research.

[23] De Craene B, Berx G (2013). Regulatory networks defining EMT during cancer initiation and progression. Nature reviews Cancer.

[24] Karnoub AE, Dash AB, Vo AP, Sullivan A, Brooks MW, Bell GW, Richardson AL, Polyak K, Tubo R, Weinberg RA (2007). Mesenchymal stem cells within tumour stroma promote breast cancer metastasis. Nature.

[25] Ma XJ, Dahiya S, Richardson E, Erlander M, Sgroi DC (2009). Gene expression profiling of the tumor microenvironment during breast cancer progression. Breast cancer research : BCR.

[26] Taube JH, Herschkowitz JI, Komurov K, Zhou AY, Gupta S, Yang J, Hartwell K, Onder TT, Gupta PB, Evans KW, Hollier BG, Ram PT, Lander ES, Rosen JM, Weinberg RA, Mani SA (2010). Core epithelial-to-mesenchymal transition interactome gene-expression signature is associated with claudin-low and metaplastic breast cancer subtypes. Proceedings of the National Academy of Sciences of the United States of America.

[27] Mani SA, Guo W, Liao MJ, Eaton EN, Ayyanan A, Zhou AY, Brooks M, Reinhard F, Zhang CC, Shipitsin M, Campbell LL, Polyak K, Brisken C, Yang J, Weinberg RA (2008). The epithelial-mesenchymal transition generates cells with properties of stem cells. Cell.

[28] Elenbaas B, Spirio L, Koerner F, Fleming MD, Zimonjic DB, Donaher JL, Popescu NC, Hahn WC, Weinberg RA (2001). Human breast cancer cells generated by oncogenic transformation of primary mammary epithelial cells. Genes & development.

[29] Seewaldt V (2014). ECM stiffness paves the way for tumor cells. Nature medicine.

[30] Bradham DM, Igarashi A, Potter RL, Grotendorst GR (1991). Connective tissue growth factor: a cysteine-rich mitogen secreted by human vascular endothelial cells is related to the SRC-induced immediate early gene product CEF-10. The Journal of cell biology.

[31] Chen CC, Lau LF (2009). Functions and mechanisms of action of CCN matricellular proteins. The international journal of biochemistry & cell biology.

[32] Jun JI, Lau LF (2011). Taking aim at the extracellular matrix: CCN proteins as emerging therapeutic targets. Nature reviews Drug discovery.

[33] Hall-Glenn F, Lyons KM (2011). Roles for CCN2 in normal physiological processes. Cellular and molecular life sciences : CMLS.

[34] Dornhofer N, Spong S, Bennewith K, Salim A, Klaus S, Kambham N, Wong C, Kaper F, Sutphin P, Nacamuli R, Hockel M, Le Q, Longaker M, Yang G, Koong A, Giaccia A (2006). Connective tissue growth factor-specific monoclonal antibody therapy inhibits pancreatic tumor growth and metastasis. Cancer research.

[35] Yamamoto Y, Gaynor RB (2001). Therapeutic potential of inhibition of the NF-kappaB pathway in the treatment of inflammation and cancer. The Journal of clinical investigation.

[36] Burstein B, Maguy A, Clement R, Gosselin H, Poulin F, Ethier N, Tardif JC, Hebert TE, Calderone A, Nattel S (2007). Effects of resveratrol (trans-3,5,4′-trihydroxystilbene) treatment on cardiac remodeling following myocardial infarction. The Journal of pharmacology and experimental therapeutics.

[37] Sonnylal S, Xu S, Jones H, Tam A, Sreeram VR, Ponticos M, Norman J, Agrawal P, Abraham D, de Crombrugghe B (2013). Connective tissue growth factor causes EMT-like cell fate changes *in vivo* and *in vitro*. Journal of cell science.

[38] Thiery JP (2002). Epithelial-mesenchymal transitions in tumour progression. Nature reviews Cancer.

[39] Yang J, Weinberg RA (2008). Epithelial-mesenchymal transition: at the crossroads of development and tumor metastasis. Developmental cell.

[40] Lipson KE, Wong C, Teng Y, Spong S (2012). CTGF is a central mediator of tissue remodeling and fibrosis and its inhibition can reverse the process of fibrosis. Fibrogenesis & tissue repair.

[41] Chen PS, Wang MY, Wu SN, Su JL, Hong CC, Chuang SE, Chen MW, Hua KT, Wu YL, Cha ST, Babu MS, Chen CN, Lee PH, Chang KJ, Kuo ML (2007). CTGF enhances the motility of breast cancer cells via an integrin-alphavbeta3-ERK1/2-dependent S100A4-upregulated pathway. Journal of cell science.

[42] Julien S, Puig I, Caretti E, Bonaventure J, Nelles L, van Roy F, Dargemont C, de Herreros AG, Bellacosa A, Larue L (2007). Activation of NF-kappaB by Akt upregulates Snail expression and induces epithelium mesenchyme transition. Oncogene.

[43] Tran CM, Shapiro IM, Risbud MV (2013). Molecular regulation of CCN2 in the intervertebral disc: lessons learned from other connective tissues. Matrix biology : journal of the International Society for Matrix Biology.

[44] Elo TD, Valve EM, Seppanen JA, Vuorikoski HJ, Makela SI, Poutanen M, Kujala PM, Harkonen PL (2010). Stromal activation associated with development of prostate cancer in prostate-targeted fibroblast growth factor 8b transgenic mice. Neoplasia.

[45] Cox TR, Erler JT (2011). Remodeling and homeostasis of the extracellular matrix: implications for fibrotic diseases and cancer. Disease models & mechanisms.

[46] Zhang K, Corsa CA, Ponik SM, Prior JL, Piwnica-Worms D, Eliceiri KW, Keely PJ, Longmore GD (2013). The collagen receptor discoidin domain receptor 2 stabilizes SNAIL1 to facilitate breast cancer metastasis. Nature cell biology.

[47] Curtis C, Shah SP, Chin SF, Turashvili G, Rueda OM, Dunning MJ, Speed D, Lynch AG, Samarajiwa S, Yuan Y, Graf S, Ha G, Haffari G, Bashashati A, Russell R, McKinney S (2012). The genomic and transcriptomic architecture of 2,000 breast tumours reveals novel subgroups. Nature.

[48] Rivas MA, Carnevale RP, Proietti CJ, Rosemblit C, Beguelin W, Salatino M, Charreau EH, Frahm I, Sapia S, Brouckaert P, Elizalde PV, Schillaci R (2008). TNF alpha acting on TNFR1 promotes breast cancer growth via p42/P44 MAPK, JNK, Akt and NF-kappa B-dependent pathways. Experimental cell research.

[49] Calzascia T, Pellegrini M, Hall H, Sabbagh L, Ono N, Elford AR, Mak TW, Ohashi PS (2007). TNF-alpha is critical for antitumor but not antiviral T cell immunity in mice. The Journal of clinical investigation.

[50] Shats I, Gatza ML, Chang JT, Mori S, Wang J, Rich J, Nevins JR (2011). Using a stem cell-based signature to guide therapeutic selection in cancer. Cancer research.

[51] Jacobson A, Cunningham JL (2012). Connective tissue growth factor in tumor pathogenesis. Fibrogenesis & tissue repair.

[52] Cunningham JL, Tsolakis AV, Jacobson A, Janson ET (2010). Connective tissue growth factor expression in endocrine tumors is associated with high stromal expression of alpha-smooth muscle actin. European journal of endocrinology / European Federation of Endocrine Societies.

[53] Zhao H, Jin G, Cui K, Ren D, Liu T, Chen P, Wong S, Li F, Fan Y, Rodriguez A, Chang J, Wong ST (2013). Novel modeling of cancer cell signaling pathways enables systematic drug repositioning for distinct breast cancer metastases. Cancer research.

[54] Andersen JN, Sathyanarayanan S, Di Bacco A, Chi A, Zhang T, Chen AH, Dolinski B, Kraus M, Roberts B, Arthur W, Klinghoffer RA, Gargano D, Li L, Feldman I, Lynch B, Rush J Pathway-based identification of biomarkers for targeted therapeutics: personalized oncology with PI3K pathway inhibitors. Science translational medicine.

[55] Cheng J, Zhu X, Cheng H, Zhao H, Wong ST (2013). A quantitative analysis of F-actin features and distribution in fluorescence microscopy images to distinguish cells with different modes of motility. Conference proceedings : Annual International Conference of the IEEE Engineering in Medicine and Biology Society IEEE Engineering in Medicine and Biology Society Annual Conference.

