# Supplementary material for: Epithelial derived CTGF promotes breast tumor progression via inducing EMT and collagen I fibers deposition
Source: Oncotarget. 2015 Jul 29;6(28):25320–38. doi: 10.18632/oncotarget.4659 (PMC4694834; doi:10.18632/oncotarget.4659)
Supplement: Supplementary file 1 [file oncotarget-06-25320-s001.pdf]

SUPPLEMENTARY MATERIALS

PRIMERS FOR QRT-PCR ANALYSIS

CTGF (5′-AAGACCTGTGGGATGGGC-3′ and 5′-TGGTGCAGCCAGAAAGCTC-3′)

CK18 (5′-CCAGTCTGTGGAGAACGACA-3′ and 5′-ATCTGGGCTTGTAGGCCTTT-3′)

CDH1 (5′-TGCCCAGAAAATGAAAAAGG-3′ and 5′-GTGTATGTGGCAATGCGTTC-3′)

CDH2 (5′-ACAGTGGCCACCTACAAAGG-3′ and 5′-CCGAGATGGGGTTGATAATG-3′)

FN1 (5′-CAGTGGGAGACCTCGAGAAG-3′ and 5′-TCCCTCGGAACATCAGAAAC-3′)

Vim (5′-GAGAACTTTGCCGTTGAAGC-3′ and 5′-GCTTCCTGTAGGTGGCAATC-3′)

Primary antibodies for western blot analysis

anti-E-Cadherin (Invitrogen, Catalog No. 33–4000); anti-N-Cadherin (BD Transduction Laboratories, Catalog No 610920); anti-fibronectin (BD Transduction Laboratories, Catalog No 610077); anti-Phospho-IKKα/β (Cell signaling, Catalog No. 2697); anti-IKKα/β (Santa cruz, catalog No. sc-7607); anti-Vimentin (Millipore, catalog No. AB-1620); anti-Cytokeratin 18 (Abcam, catalog No. ab82254); anti-CTGF (Abcam, catalog No. ab6992); anti-β-actin (Sigma, catalog No. A2228).

SUPPLEMENTARY FIGURES AND TABLES

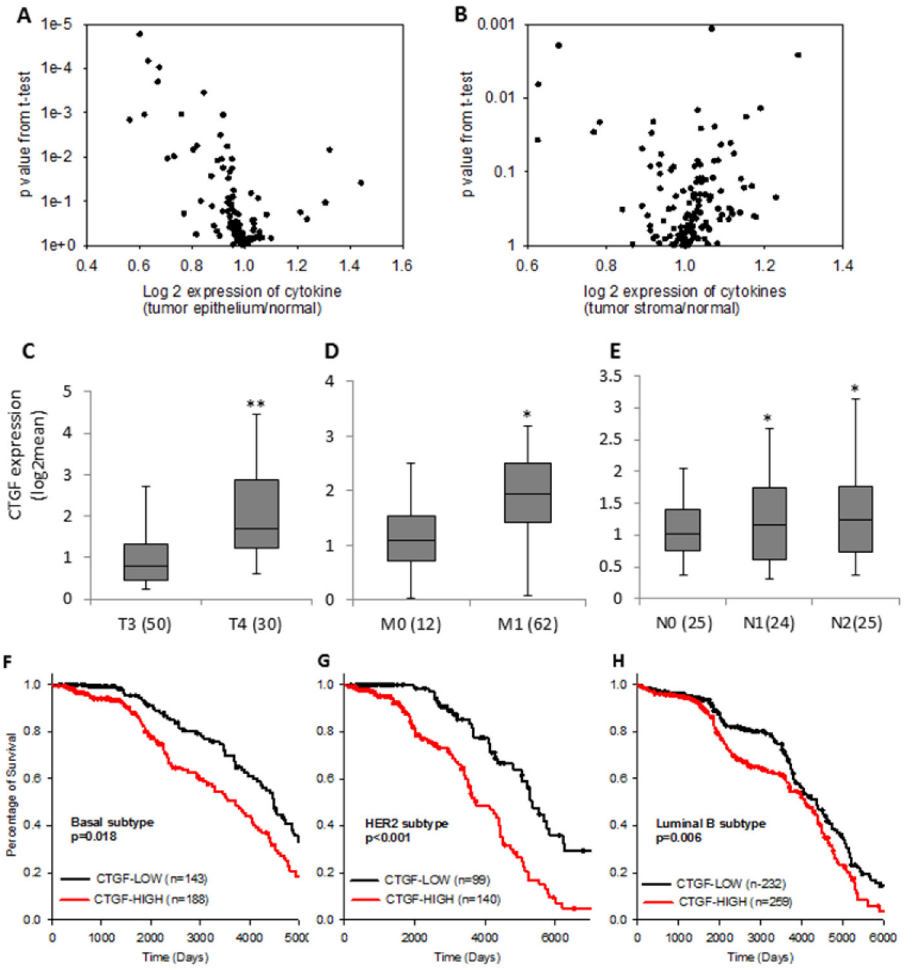

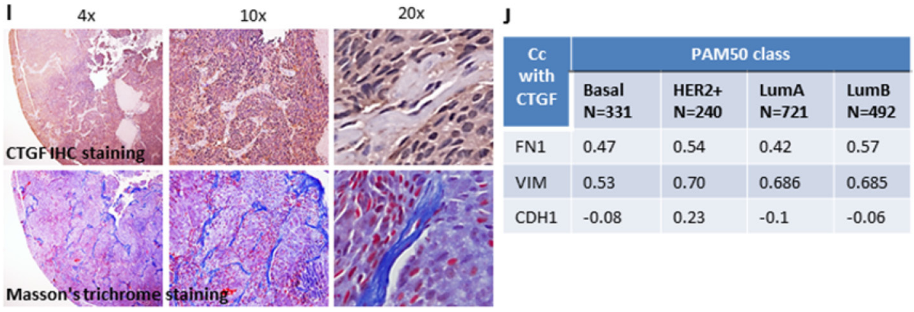

J

Cc with CTGF	PAM50 class			
	Basal N=331	HER2+ N=240	LumA N=721	LumB N=492
FN1	0.47	0.54	0.42	0.57
VIM	0.53	0.70	0.686	0.685
CDH1	-0.08	0.23	-0.1	-0.06

Supplementary Figure S1: A–B. Volcano plots showing the expression of the 132 cytokines in the tumor epithelium and tumor-stroma specimens (invasive vs. normal+ ductal carcinoma *in situ*). **C–E.** High expression of *CTGF* in the bulk tumor specimens correlated with advanced TNM stages in a 167 breast tumor cohort (GSE4382). Sample numbers were listed in the brackets. **F–H.** High expression of *CTGF* in the breast tumor subtypes correlated with poor overall survival in the Curtis Breast cohort. PAM50 classification was used to define subtypes. Sample numbers were listed in the brackets. **I.** Representative images showing the immunohistological staining of CTGF and Masson's trichrome staining of tumor stroma in the PDX tumor sections. **J.** Correlation of the expressions between *CTGF* and EMT markers in breast tumor subtypes in the Curtis Breast cohort.

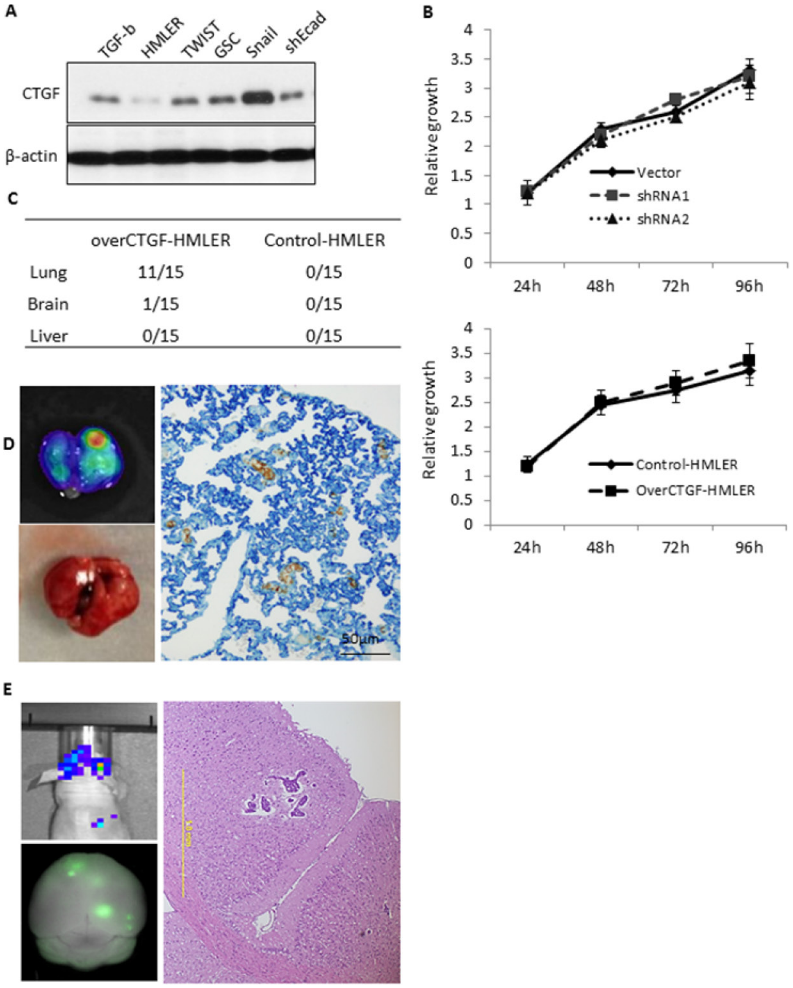

Supplementary Figure S2: A. Western blot analysis of CTGF in the series HMLER cells. **B.** *In vitro* proliferation of the indicated cell lines. **C.** Spontaneous metastasis of the over-CTGF-HMLER tumor cell xenografts. **D.** Representative bioluminescent image (upper left), and immunohistological staining of human CD44/mouse CD34 (right) image showing the lung metastasis of the over-CTGF-HMLER tumor cell xenograft. **E.** Representative bioluminescent image (upper left), GFP fluorescent image (lower left), and H & E staining image showing the brain metastasis of the over-CTGF-HMLER tumor cell xenograft.

A

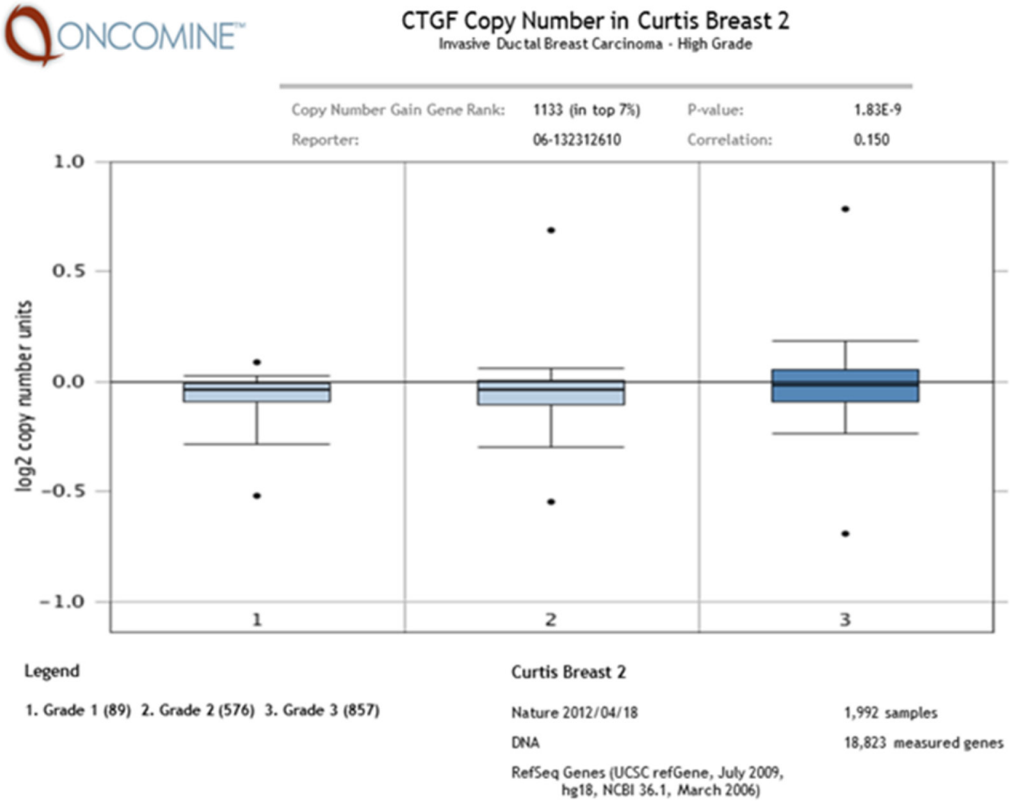

Supplementary Figure S3: CTGF copy number gain correlates with advanced tumor grades in the Curtis breast cohort (*n* = 1992).

Supplementary Table S1: Expression of 132 cytokines, chemokines, and growth factors in patient-matched tumor epithelium specimens from 38 breast carcinoma and 28 normal breast samples

Supplementary Table S2: Expression of 132 cytokines, chemokines, and growth factors in patient-matched tumor-stroma specimens from 38 breast carcinoma and 28 normal breast samples
